# Rapid genome resequencing of an atoxigenic strain of *Aspergillus carbonarius*

**DOI:** 10.1038/srep09086

**Published:** 2015-03-13

**Authors:** F. Javier Cabañes, Walter Sanseverino, Gemma Castellá, M. Rosa Bragulat, Riccardo Aiese Cigliano, Armand Sánchez

**Affiliations:** 1 Veterinary Mycology Group, Department of Animal Health and Anatomy, Universitat Autònoma de Barcelona, Bellaterra, Catalonia, Spain; 2 Sequentia Biotech SL, Barcelona, Catalonia, Spain; 3 Departament de Genètica Animal, Centre de Recerca en AgriGenòmica (CRAG), CSIC-IRTA-UAB-UB, Universitat Autònoma de Barcelona, Bellaterra, Catalonia, Spain

## Abstract

In microorganisms, Ion Torrent sequencing technology has been proved to be useful in whole-genome sequencing of bacterial genomes (5 Mbp). In our study, for the first time we used this technology to perform a resequencing approach in a whole fungal genome (36 Mbp), a non-ochratoxin A producing strain of *Aspergillus carbonarius*. Ochratoxin A (OTA) is a potent nephrotoxin which is found mainly in cereals and their products, but it also occurs in a variety of common foods and beverages. Due to the fact that this strain does not produce OTA, we focused some of the bioinformatics analyses in genes involved in OTA biosynthesis, using a reference genome of an OTA producing strain of the same species. This study revealed that in the atoxigenic strain there is a high accumulation of nonsense and missense mutations in several genes. Importantly, a two fold increase in gene mutation ratio was observed in PKS and NRPS encoding genes which are suggested to be involved in OTA biosynthesis.

Ochratoxin A (OTA) is a potent nephrotoxin which is found mainly in cereals and their products, but it also occurs in a variety of common foods and beverages such chocolate, dried fruits, coffee or wine. This mycotoxin is produced by several species of *Penicillium* and *Aspergillus* among which *Aspergillus carbonarius* is the main responsible source of this mycotoxin in wine, grapes and dried vine fruits from main viticultural regions worldwide^1^.

The biosynthetic pathway of other mycotoxins has been elucidated, as it is the case of the aflatoxin biosynthetic gene cluster of *Aspergillus flavus*, largely responsible for the aflatoxin contamination of agricultural crops^2^. However, little is known about the genes involved in OTA biosynthesis. OTA consists of a polyketide derived chlorinated-dihydromethyl-isocoumarin linked to phenylalanine. Different polyketide synthase (PKS) encoding genes involved in OTA biosynthesis have been identified in various ochratoxin A producing species^3^,^4^,^5^,^6^,^7^. To date, only a PKS gene involved in the initial steps of the OTA biosynthetic pathway and a nonribosomal peptide synthetase (NRPS) gene have been related to this biosynthetic gene cluster in *A. carbonarius*^8^,^9^. Recently, the genome sequence of an OTA producer strain of *A. carbonarius* ITEM 5010 (Acv3) has been generated by the United States Department of Energy's Joint Genome Institute using the 454 and the Sanger sequencing technologies (http://jgi.doe.gov/carbonarius/)^10^. Similar to many other ascomycetes so far sequenced, the *A*. *carbonarius* genome contains a large number of PKS and NRPS encoding genes. These genes encode complex and multifunctional proteins involved in the biosynthesis of most of the fungal secondary metabolites. Sequencing technologies may ease the study of the genetic biosynthetic pathways of important mycotoxins, such as OTA. Ion Torrent sequencing technology has been proved to be useful in whole-genome sequencing of small sized genomes, up to 5 Mbp, such as some bacterial genomes^11^,^12^.

In this study, for the first time, we used the Ion Torrent technology to resequence the genome of a mold, an atoxigenic wild strain of *A. carbonarius* with a genome size of about 36 Mbp, using the reference genome Acv3 of a toxigenic *A. carbonarius* strain. Besides this main objective, and due to the fact that this strain does not produce OTA, we focused some of the bioinformatics analyses in genes involved in OTA biosynthesis.

## Results

### Detection of the OTA production in the *A. carbonarius* strains studied

**Figure 1:**
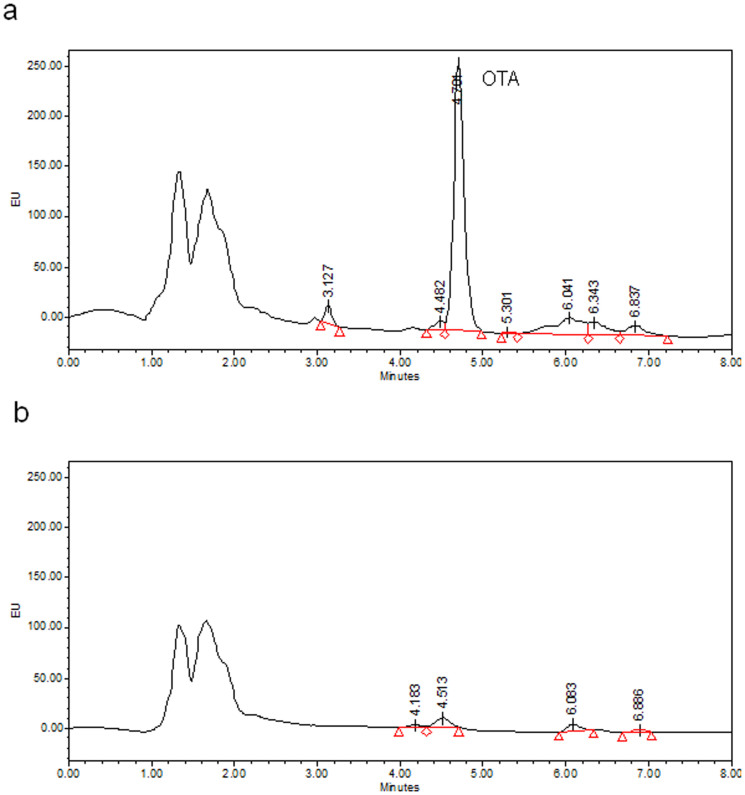
Selected chromatograms of fungal extracts analysed using HPLC coupled to a fluorescence detector of (a) the OTA producing strain of *A. carbonarius* ITEM 5010 (OTA retention time: 4.701 min) and (b) the atoxigenic strain of *A. carbonarius* A-2160, after incubation at 15°C for 7 days on Czapek Yeast extract Agar.

*Aspergillus carbonarius* strains grew in CYA medium at 15°C, 25°C and 30°C. Both strains presented good growth with proper sporulation forming typical black colonies (Figure S1). However, the non-OTA producing strain (A-2160) was not able to produce OTA at these temperatures after 7 or 15 days of incubation. On the contrary, *A. carbonarius* ITEM 5010 produced OTA at detectable levels at the three temperatures of incubation tested. This strain produced higher amounts of OTA at 15°C after 15 days of incubation than in the rest of the temperatures and incubation times tested. Figure 1 shows some selected chromatograms of the fungal strains analysed in this study with HPLC. Extracts of *A. carbonarius* ITEM 5010 (Figure 1a) presented a clear peak with the same retention time of OTA (4.7 minutes). The extracts of *A. carbonarius* A-2160 (Figure 1b) showed no signals at the same retention time of OTA.

### Resequencing study

The general resequencing genome data of the atoxigenic strain A-2160 of *A. carbonarius* is summarized in Table 1. A single Ion Torrent run was performed to resequence the genome of this strain. A total of 3,158,689 reads were sequenced for a total of 547.56 Mbp. The alignment of the reads to the Acv3 showed that more than the 84% of the no-repetitive reference genome was covered with an average depth of ~12×.

The comparisons between the multiple and unique mapping reads showed that the two strains share about 3 Mb of repetitive regions and that have a similarity higher than 97.2%. The deletions accumulated in the atoxigenic strain of *A. carbonarius* summed to ~1 Mbp and the total size of the unmapped reads was of ~39 Mb (7.75% of the reads produced by the Ion Torrent run). The alignment between the reads produced from the atoxigenic strain and the Acv3 were used to study the variation between the two strains and to highlight the genomics differences that might explain the phenotype of the atoxigenic strain.

### Single-nucleotide and deletion-insertion polymorphisms analyses

**Figure 2:**
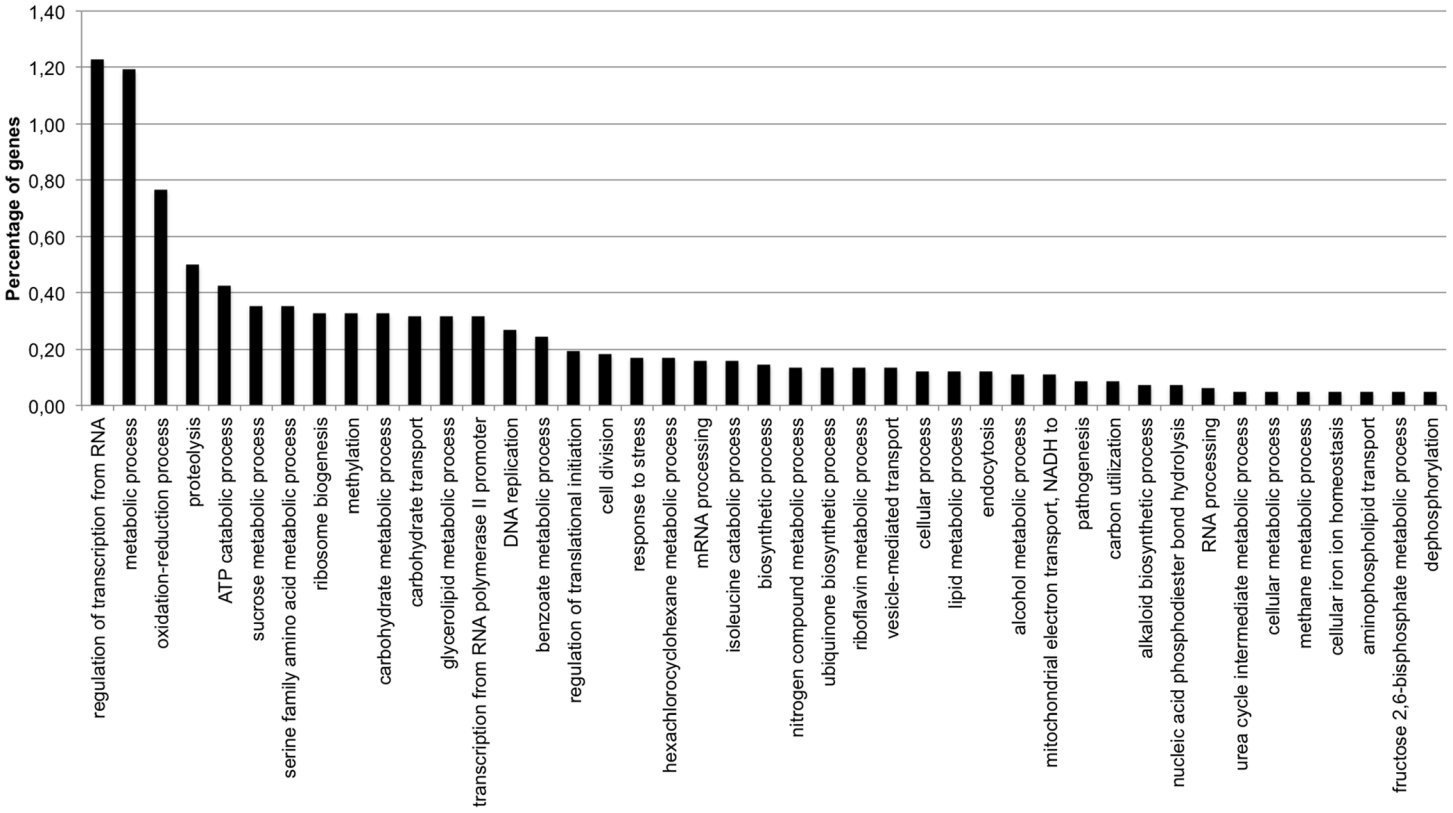
Gene Ontology (GO) families that have more than the 5% of mutated genes. In the Y axis there is the percentage of gene respect the total number of genes and in the X axis the enriched families.

All uniquely mapped regions were used to perform a specific single-nucleotide polymorphisms (SNPs) and deletion-insertion polymorphisms (DIPs) calling. A total of 52661 high quality SNPs and 7567 high quality DIPs have been identified. Due to the homozygous nature of the re-sequenced genome, all the heterozygous mutations have been discarded. High quality SNPs and DIPs have been used to perform a mutation mining according with the information of the gene annotation of the reference genome Acv3. Table 2 shows the presence of 7183 missense, 88 nonsense and 528 frameshift mutations. The missense mutations affect 3880 genes. A total of 43 Gene Ontology (GO) families have more than the 5% of mutated genes (Figure 2 and Supplementary Table S1).

### Copy number variation analysis

Using a read depth approach on the multiple mapping alignment, several copy number variation (CNV) regions were identified. The analysis showed that there were not significant duplicated regions but there were 55 deleted regions (Supplementary Table S2) containing 291 genes that fall in 60 GO classes (Supplementary Table S3). Of that, kinase regulator activity (26.57), serine-type carboxypeptidase activity (10.63) and metal cluster binding (7.97) classes have a high enrichment level. Several small scaffolds in the reference genome Acv3 resulted completely deleted.

### PKS and NRPS encoding gene analyses

**Figure 3:**
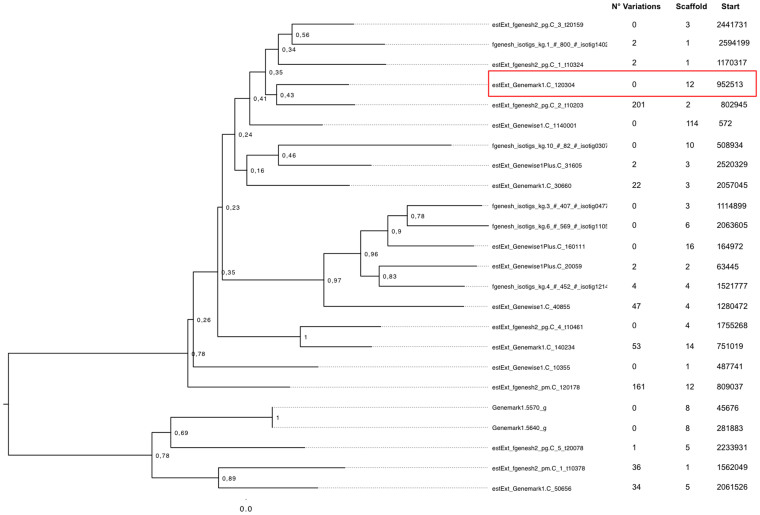
Phylogenetic reconstruction of the NRPS genes annotated on the reference genome of *A. carbonarius* (in red the NRPS estExt_Genemark1.C_120304 (PI 132610) gene).

**Figure 4:**
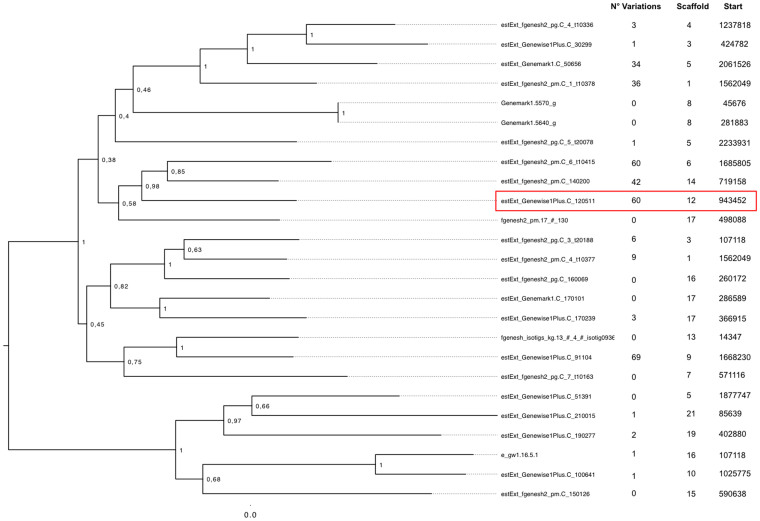
Phylogenetic reconstruction of the PKS genes annotated on the reference genome of *A. carbonarius* (in red the PKS estExt_Genewise1Plus.C_120511 (PI 173482) gene).

A total of 24 NRPS genes and 25 PKS genes were retrieved with an approach based on Blast, Inteproscan and AntiSmash. For both families a phylogenic approach was combined with the SNPs, DIPs and CNVs analyses in order to extract supplementary information about these two gene families (Figures 3 and 4). Many NRPS and PKS genes had mutations and one NRPS gene (estExt_fgenesh2_pg.C_3_t20159) and one PKS gene (estExt_Genemark1.C_50656) were deleted in the atoxigenic strain.

By using the SNPs information, we calculated the gene mutation ratio (GMR) of the entire set of genes and we compared it with the GMR of the NRPS and PKS gene families. The global GMR was of 1.1 (1 SNP each 909 bp) while the GMR of NRPS and PKS was of 2.2 (1 SNP every 454 bp) and 1.6 (1 SNP every 625 bp), respectively. Moreover the phylogenic analysis of the two gene families showed that the nearest gene (estExt_fgenesh2_pg.C_2_t10203) to the functional NRPS gene described by Gallo et al.^8^ (estExt_Genemark1.C_120304: PI 132610) was the most mutated gene in the genome with 201 mutated nucleotides (Figure 3).

In order to confirm some of these mutations by PCR techniques we focused our study in one NRPS gene (PI 132610) and one PKS gene (PI 173482) that have been reported to be involved in the OTA biosynthetic pathway of *A. carbonarius*^8^,^9^. In the NRPS gene estExt_Genemark1.C_120304 (PI 132610) we observed deletions in the promoter and in the exon 2. In order to confirm these deletions, we designed the primers pairs NRPS1F/NRPS1R and NRPS2F/NRPS2R, respectively. With primer pairs NRPS1F/NRPS1R and NRPS2F/NRPS2R, fragments of 640 bp and 671 bp were obtained, respectively. Sequences obtained were compared with those of Acv3 reference genome and no differences were observed.

**Figure 5:**
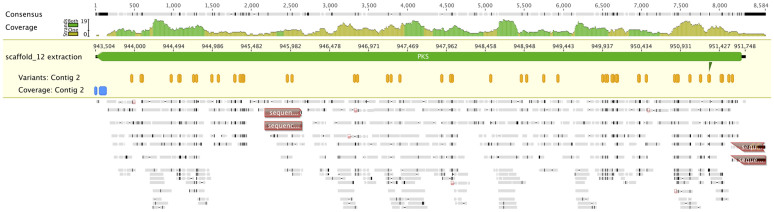
SNPs visualization of the PKS estExt_Genewise1Plus.C_120511 (PI 173482) gene. This figure shows the PKS locus extracted from the scaffold_12, where it is located, as a green arrow. The direction of the arrow shows that the gene is on the minus strand of the genome. The numbers above the arrow indicate the coordinates of the locus respect to the scaffold. The “Consensus” row shows the positions were no difference between the reference genome and the reads could be detected in grey, the SNPs in black and deletions as narrow grey lines. The numbers above the “Consensus” row refer to the coordinates respect to the gene. The “Coverage” row shows a histogram with the distribution of the reads along the gene body, the scale being from 0 to 19 reads. The “Variants: Contig 2” row shows the positions of the SNPs between the reference genome and the reads as yellow hexagons. The “Coverage: Contig 2” row shows the position of the deletions detected by the coverage approach as blue shapes. The bottom part of the figure shows the reads mapped on PKS. Reads portions that are equal to the reference are showed in grey, while SNPs and InDels are showed in black. Red boxes show the positions of sequencing primers.

**Figure 6:**
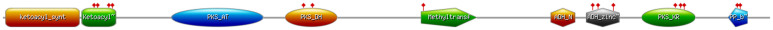
Schematic representation of the domain composition of the PKS estExt_Genewise1Plus.C_120511 (PI 173482) protein. Different domains are represented with different shapes and colors, with the relative name reported. Uncoiled regions are represented by a grey line. The identified SNPs that are affecting amino acids located in the predicted domains are reported as red marks.

In the PKS gene estExt_Genewise1Plus.C_120511 (PI 173482) we observed 24 SNPs. Among these, 7 were showed to produce missense mutations. Moreover, deletions in the first exon and in the middle of the exon 7 were identified (Figure 5). The position of the SNPs in the gene were compared with the positions of the predicted protein domains to visualize their possible effects. 15 SNPs affected 6 out of the 9 domains (Figure 6). To confirm these deletions, we designed primers pairs PKS1F/PKS1R and PKS2F/PKS2R, respectively. With primer pair PKS1F/PKS1R, a fragment of 437 bp was obtained. When the sequence was compared to Acv3, the sequence of the atoxigenic strain showed insertions (14 bp), and substitutions (7 transitions and 2 transversions). With the primer pair PKS2F/PKS2R, a fragment of 480 bp was obtained and showed 2 transitions and 2 transversions in the exon 7 compared with the sequence of the toxigenic strain Acv3.

## Discussion

Despite a large amount of data is produced with the *de novo* genomes projects the real challenge of genomics, to date, is to determine the genetic differences among individuals and to understand their relationships to the phenotypic differences within species. A possible approach is to identify the relatively small changes between a genome and a reference sequence. Many sequencing technologies have been developed for resequencing projects, but few of them are suitable to study variation between individuals or species. Usually, sequencers that produce small reads with a high depth of coverage and are able to sequence with a pair-end technology are a good choice to perform resequencing studies. Unfortunately, the cost of these experiments is often not affordable by small research groups. Other cheap and accessible sequencers are available in the market and could be used for this purpose.

In the present study, for the first time, we used a single shotgun run of the 318 Ion Torrent chip to perform a comparison between the reference genome Acv3 and an atoxigenic wild strain of the same species. We tried to elucidate specific genes involved in OTA biosynthesis comparing the reference genome of an OTA-producing strain of *A carbonarius* (Acv3) against a not sequenced genome of a non OTA-producing wild strain of the same species. Previous work on Acv3 revealed that the genome encodes at least 24 putative NRPS and 21 PKS encoding genes^8^,^9^. In our study a total of 24 NRPS genes and 25 PKS genes were retrieved. We also detected that in the atoxigenic strain there was a high accumulation of nonsense and missense mutations in several genes such as PKS and NRPS encoding genes. The high mutation rate of these genes could explain the lack of production of OTA by the atoxigenic strain.

OTA is derived from the fungal polyketide biosynthetic pathway. Main parts of the OTA molecule are an isocoumarin moiety and the amino acid phenylalanine. However, the molecular basis of OTA biosynthesis is poorly understood. The structure of OTA suggests a biosynthetic pathway including various enzymatic steps. Besides the biosynthesis of phenylalanine, other enzimatic activities are required in the OTA pathway such as a PKS for the synthesis of the polyketide dihydroisocoumarin, a chlorinating enzyme, a methylase, an esterase and a NRPS for ligation of the phenylalanine to the dihydroisocoumarin. So far only some OTA related PKS genes have been detected in OTA producing species such as *Aspergillus ochraceus*^3^, *Aspergillus westerdijkiae*^6^, *Penicillium nordicum*^4^, *Penicillium verrucosum*^7^, *Aspergillus niger*^13^ and *A. carbonarius*^9^.

Nowadays, little is known about the genes involved in the OTA biosynthesis of *A. carbonarius* which is a consistent OTA-producing species. However recently, a few wild non OTA-producing strains of *A. carbonarius* have been discovered^14^ and some nonochratoxigenic mutant strains have been obtained both by the inactivation of genes encoding a NRPS^8^ and a PKS^9^ in a wild type OTA-producing strain of *A.* *carbonarius*, giving interesting new insights in the biosynthetic pathway of this mycotoxin. While no differences were observed in the sequences of this NRPS gene, several SNP were found in the PKS gene of the atoxigenic strain. Both genes^8^,^9^ have been described to be involved in the OTA biosynthetic pathway of *A. carbonarius*. Changes detected in the PKS gene could affect the function of the protein and may be responsible for atoxigenicity in the non OTA-producing strain of *A. carbonarius*.

With our findings we demonstrate that Ion Torrent technology could be a good alternative to more expensive ones and that thanks to a specific bioinformatics pipeline it is possible to extract useful information. It seems clear that the sensitive point is to develop a specific workflow not only to manage and filter data to reach a high quality of the results but also to take advantage of single-end runs to analyze the structural variations between two genomes^15^,^16^. In this case a homemade pipeline was developed to reach high standards of variation calling using the newest published algorithms as well as specific bioinformatics methods. For the SNPs and DIPs calling we modified our pipeline, SUPERW (**S**imply **U**nified **P**air-**E**nd **R**ead **W**orkflow), to work with single-end reads. Being not possible to use a pair-read approach, the SVs calling was performed by using a copy number variation approach, based on the differences in depth of coverage.

In order to contribute to elucidate the little-known OTA biosynthetic gene cluster of *A. carbonarius*, we plan to investigate the role of the mutations detected in PKS and NRPS encoding genes in this study. In the same direction, we plan also further metabolomic studies of both toxigenic and atoxigenic strains.

In conclusion, a deep *in silico* approach based on a comparative genomic study revealed that in the atoxigenic strain there is a high accumulation of nonsense and missense mutations in several genes. Importantly, a two fold increase in GMR was observed in PKS and NRPS encoding genes thus providing interesting clues on their role in OTA biosynthetic pathway.

## Methods

### OTA production ability detection

OTA production was confirmed using a previously described high-pressure liquid chromatography (HPLC) screening method^17^ designed in our laboratory. The strains A-1796 (kindly provided by P. Battilani as W-201 ( = ITEM 5010) and A-2160 ( = CECT 20837) from our fungal collection were first three point inoculated on Czapek Yeast extract Agar (CYA) and incubated at 15, 25 and 30°C. After 7 and 15 days of incubation at each temperature assayed and from each strain, three agar plugs were removed from different points of the colony and extracted with 0.5 ml of methanol. The extracts were filtered and maintained at 4°C until their analysis. Two replicates for each strain and incubation condition assayed were used. The detection limit of the extraction procedure and the HPLC technique was 0.02 ng OTA, and the limit of quantification of the HPLC technique with the extraction procedure was 0.01 μg/g for this mycotoxin.

### DNA preparation and gene sequencing analysis

The non OTA-producing strain of *A. carbonarius* A-2160 was grown on malt extract broth medium in the dark at 25°C for 48 h. Mycelium was recovered and grounded into fine powder using a mortar and pestle after brief nitrogen deep freezing. DNA extraction was carried out using phenol–chloroform according to the protocol described by Bragulat et al.^18^ and treated with RNase to degrade RNA. Primers for partial amplification of NRPS estExt_Genemark1.C_120304 (PI 132610) and PKS estExt_Genewise1Plus.C_120511 (PI 173482) were designed with Primer3 tool^19^. Primers NRPS1F (5′–TTTCCCCTACTTCGTGCCAC-3′) and NRPS1R (5′-CTAGAACCCCGTGCCCTTTT-3′) and NRPS2F (5′-GTGGTTGTGTCTCCGGATGT-3′) and NRPS2R (5′-CCGCTCCTCATCATGCAGAT-3′) were designed to confirm the deletion in the promoter and in the exon 2 of NRPS estExt_Genemark1.C_120304 (PI 132610) gene, respectively. Primers PKS1F (5′-GACAAGGGTGGTGAGGATGG-3′) and PKS1R (5′- CATTGTGCTGGACTTTGGGC-3′) and PKS2F (5′-CAACTTCCTCCTGACCGCAT-3′) and PKS2R (5′-CCCACTGCACGGATCTGAAT-3′) were designed to confirm the deletion in the first exon and in the middle of exon 7 of PKS estExt_Genewise1Plus.C_120511 (PI 173482) gene, respectively. The PCR products were purified with MultiScreen filter plates (Millipore, Barcelona, Spain) following the manufacturer's protocol. The purified product was used as a template for sequencing. The Big-Dye Terminator v3.1 Cycle Sequencing kit (Applied Biosystems) and primers NRPS1F/R, NRPS2F/R, PKS1F/R and PKS2F/R were used for sequencing as specified by the manufacturer. An Applied Biosystems 3730 sequencer was employed to obtain the DNA sequences. Sequence alignments were performed using the software program Clustal X v2.0.12^20^.

### DNA genome Ion Torrent sequencing

Fragment DNA library construction was performed using the Ion Xpress Plus Fragment Library Kit (Life Technologies). 100 ng of genomic DNA was used for the enzymatic shearing and ligation of Ion Torrent™ adapters according to the manufacturer's instructions. Fragments of 330 bp were selected by 2% agarose gel electrophoresis using the E-Gel iBase™ Power System and E-Gel Safe Imager™ (Invitrogen, Life Technologies). The quality and quantity of the genomic DNA library was assessed by analysing them in High Sensitivity DNA chip in the 2100 Bioanalyzer (Agilent). Template preparation for sequencing was made in The Ion OneTouch™ 2 System by the Ion OneTouch 200 Template Kit v2 (Life Technologies) according to manufacturer's instructions.

Sequencing was performed on the Ion Torrent Personal Genome Machine (PGM) platform using the Ion PGM 200 Sequencing Kit and an Ion 318 chip (Life Technologies).

### Whole fungal genome resequencing

Our workflow was divided in three steps. The filtering and mapping step used as input the raw reads produced by an NGS sequencer and, utilizing the custom user parameters, automatically filters the raw reads creating a new high quality subset of reads suitable for mapping analyses^21^. After the filtering step, all the samples were mapped against the Acv3 reference genome using the bwa mem algorithms^22^. For the variation calling the mapped bam files and the reference genome are the input file of the third step of the pipeline in which the user can choose to extract small variations (SNPs and DIPs), large variations (deletions, inversions and duplications) or both^23^. More than 3E106 ion torrent raw reads with a maximum length of 328 bp were used in the resequencing experiment. The filter and trimming process made with an homemade pipeline based on cutadapt tool^24^ discarded only the 0.1% of the entire reads set and decreased the reads length at 262 bp (minimum reads length 20 bp). The alignment process was conducted on the reference genome Acv3 composed by 963 scaffolds for a total size of 36 Mb. The reads were mapped on Acv3 with the bwa-mem algorithm^22^. About 2.8 mio of reads were mapped on the reference genome, leaving 0.3 mio of unmapped reads. About 2.3 mio mapped uniquely (Supplementary Table S4). Two alignments file were created to perform the SNPs and DIP calling and CNV analysis. The first alignment was created only with the unique mapped reads and the other with all the mapped reads for the studies. Both alignments were filtered for mapping quality and PCR-duplicates were removed. In the two mapping experiment the mean coverage is respectively 12.29× and 11.36× and the unmapped region size is of 6 Mb and 3.5 Mb highlighting that the reference genome Acv3 is composed of ~3 Mb of repetitive regions.

### SNPs and DIPs calling

The calling was performed with samtools^25^ on the unique mapped alignment and resulting SNPs and DIPs were filtered and annotated with SnpSift^26^. All the homozygous SNPs > 4 and DIPs with a depth >6 and a samtools quality >30 were used for further analysis. The SNP and DIPs annotation and mining were performed with SNPeff^27^ with the creation of a custom SNPeff database of Acv3 and custom parameter for the fungi genomes. As reference genes model was used the gff3 file of the reference genome Acv3.

### CNV analysis

CNV analysis was performed with cnvnator^28^ using the multiple mapped alignment. For each scaffold the CNV regions were extracted using a bin size of 1 Kb, filtered for p-value < 0.05, duplication level <50% and divided in deleted or duplicated regions according with the coverage of the copy number variated regions (Table S5). With the coordinates of the CNVs list of duplicated or deleted genes was created.

### NRPS and PKS genes identification

In order to identify the NRPS and PKS genes in *A. carbonarius*, the protein sequences of the PKS estExt_Genewise1Plus.C_120511 and of the NRPS estExt_Genemark1.C_120304 were used for a BLASTp search. The resulting genes were filtered by using AntiSmash and Interproscan identification of domain composition. Only the NRPS genes showing the same domain composition of estExt_Genemark1.C_120304, i.e. AMP-binding (PF00501), PP-binding (PF00550), Condensation (PF00668), were used for further analyses. Similarly only the PKS genes showing the same domain composition of estExt_Genewise1Plus.C_120511, i.e. Ketoacyl-synt_C (PF02801), Acyl_transf_1 (PF00698), PS-DH (PF14765), Methyltransf_12 (PF08242), ADH_N (PF08240), KR (PF08659) and PP-binding (PF00550), were used for further analyses. The identified NRPS and PKS genes are listed in the Supplementary Table S6. Domain composition of the PKS estExt_Genewise1Plus.C_120511 (PI 173482) protein was predicted by using SMART (http://smart.embl-heidelberg.de/) looking for outlier homologues and PFAM domains. The diagram of the protein and the affecting SNPs was performed with Expasy MyDomains (http://prosite.expasy.org/mydomains).

### Molecular phylogenetic analysis

Phylogeny histories of NRPS and PKS were inferred within Phylogeny.fr environment (http://www.phylogeny.fr/ *webcite*). Each protein group was aligned with MUSCLE. Maximum Likelihood phylogeny reconstruction was employed with WAG and WAG + I + G + F models for NRPS and PKS, respectively. The bootstrap consensus trees were inferred from 100 replicates. The phylogenetic trees were visualized with FigTree 1.3.1.

### Gene Onthology analysis

The Gene Onthology analysis has been perfomed with Blast2Go v 2.6.6^29^ using the standard option and standard annotation pipeline. The results were used in REVIGO^30^ to cleaning the GO results, create a non-redundant dataset and highlight the more representative GO categories.

## Author Contributions

F.J.C., M.R.B. and G.C. designed the work; G.C. performed PCR studies; M.R.B. performed mycological and HPLC studies; A.S. coordinated genome resequencing studies; W.S. and R.A.C. performed bioinformatic studies; F.J.C. coordinated manuscript writing.

## Additional information

**Accession Codes**: The genome resequencing information of *A. carbonarius* A-2160 has been deposited in the European Nucleotide Archive (ENA) under accession PRJEB6789.

## Supplementary Material

Supplementary Information

## Figures and Tables

**Table 1:** Resequencing statistics of the atoxigenic strain of *A. carbonarius*

Total Number of Bases	547.56 Mbp
Number of Q20 Bases	405.83 Mbp
Mean length	173 bp
Longest Read	396 bp
Mean Coverage Depth (Q20)	11.36×
Total Number of reads	5,184,113
Filtered Polyclonal	1,661,202
Filtered Primer Dimer	58
Filtered Low Quality	364,164
Total Number of reads	3,158,689

**Table 2:** SNPs and DIPs annotation

SNPs effect	N°	DIPs effect	N°
Missense	7183	Codon deletion	75
STOP codon gain	88	Codon Insertion	37
Silent	11342	Frameshift	528
Other	34048	STOP codon gain	2
Total	52661	Other	6925
Mutation Rate	1/666 bp	Mutation Rate	1/4626 bp
