# Supplementary material for: Rapid genome resequencing of an atoxigenic strain of Aspergillus carbonarius
Source: Sci Rep. 2015 Mar 13;5:9086. doi: 10.1038/srep09086 (PMC4358045; doi:10.1038/srep09086)
Supplement: Supplementary Information [file srep09086-s1.pdf]

Supplementary information:

Rapid genome resequencing of an atoxigenic strain of *Aspergillus carbonarius*.

F. Javier Cabañes[*1], Walter Sanseverino[2], Gemma Castellá[1], M. Rosa Bragulat[1], Riccardo Aiese Cigliano[2] & Armand Sánchez[3].

[1]Veterinary Mycology Group, Department of Animal Health and Anatomy, Universitat Autònoma de Barcelona, Bellaterra, Catalonia, Spain

[2]Sequentia Biotech SL, Barcelona, Spain

[3]Departament de Genètica Animal, Centre de Recerca en AgriGenòmica (CRAG), CSIC-IRTA-UAB-UB, Universitat Autònoma de Barcelona, Bellaterra, Catalonia,Spain

Figure S1. Colonies of the atoxigenic strain A-2160 (a) and the toxigenic strain A-1796 (b) of *A. carbonarius* grown on Czapek Yeast extract Agar at 15ºC for 15 days.

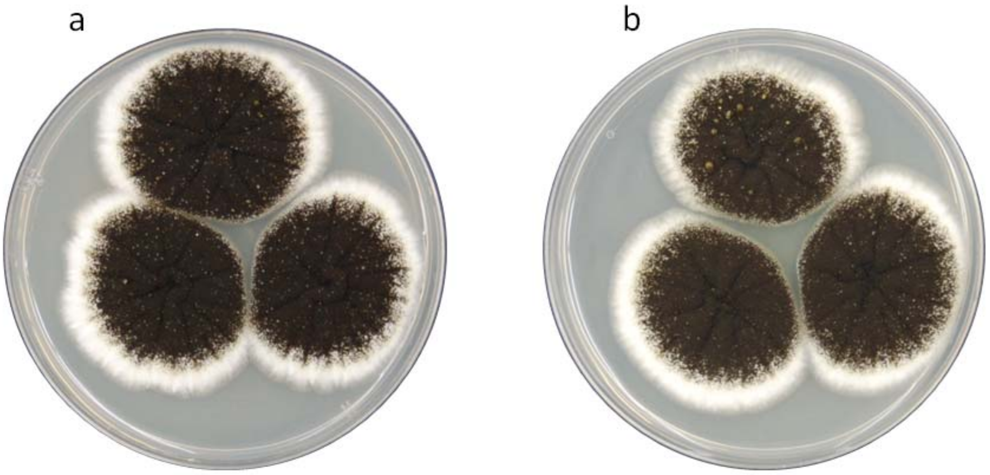

Table S1. Gene Ontology (GO) families that have more than 5% of mutated genes

GO ID	Description	Percentage of genes	GO ID	Description	Percentage of genes
GO:0006357	regulation of transcription from RNA polymerase II promoter	1,23	GO:0006807	nitrogen compound metabolic process	0,13
GO:0008152	metabolic process	1,19	GO:0006744	ubiquinone biosynthetic process	0,13
GO:0055114	oxidation-reduction process	0,77	GO:0006771	riboflavin metabolic process	0,13
GO:0006508	proteolysis	0,50	GO:0016192	vesicle-mediated transport	0,13
GO:0006200	ATP catabolic process	0,43	GO:0009987	cellular process	0,12
GO:0005985	sucrose metabolic process	0,35	GO:0006629	lipid metabolic process	0,12
GO:0009069	serine family amino acid metabolic process	0,35	GO:0006897	endocytosis	0,12
GO:0042254	ribosome biogenesis	0,33	GO:0006066	alcohol metabolic process	0,11
GO:0032259	methylation	0,33	GO:0006120	mitochondrial electron transport, NADH to ubiquinone	0,11
GO:0005975	carbohydrate metabolic process	0,33	GO:0009405	pathogenesis	0,09
GO:0008643	carbohydrate transport	0,32	GO:0015976	carbon utilization	0,09
GO:0046486	glycerolipid metabolic process	0,32	GO:0009821	alkaloid biosynthetic process	0,07
GO:0006366	transcription from RNA polymerase II promoter	0,32	GO:0090305	nucleic acid phosphodiester bond hydrolysis	0,07
GO:0006260	DNA replication	0,27	GO:0006396	RNA processing	0,06
GO:0018874	benzoate metabolic process	0,24	GO:0000051	urea cycle intermediate metabolic process	0,05
GO:0006446	regulation of translational initiation	0,19	GO:0044237	cellular metabolic process	0,05
GO:0051301	cell division	0,18	GO:0015947	methane metabolic process	0,05
GO:0006950	response to stress	0,17	GO:0006879	cellular iron ion homeostasis	0,05
GO:0019497	hexachlorocyclohexane metabolic process	0,17	GO:0015917	aminophospholipid transport	0,05
GO:0006397	mRNA processing	0,16	GO:0006003	fructose 2,6-bisphosphate metabolic process	0,05
GO:0006550	isoleucine catabolic process	0,16	GO:0016311	dephosphorylation	0,05
GO:0009058	biosynthetic process	0,15			

Table S2. Deletions found in the *A. carbonarius* atoxic strain containing 291 genes

Scaffold	Start Coordinate	Stop Coordinate	ID	Read Depth Value	Length
scaffold_1	706001	708000	deletion_5	0,0678696	2000
scaffold_2	1907001	1912000	deletion_22	0,0311621	5000
scaffold_3	305001	309000	deletion_32	0,137681	4000
scaffold_3	685001	688000	deletion_34	0,0479417	3000
scaffold_3	815001	837000	deletion_36	0,111913	22000
scaffold_3	1994001	1997000	deletion_43	0,0462044	3000
scaffold_3	2421001	2459000	deletion_44	0,106535	38000
scaffold_4	33001	325000	deletion_45	0,427008	292000
scaffold_5	2047001	2078000	deletion_65	0,212864	31000
scaffold_6	64001	281000	deletion_68	0,388807	217000
scaffold_6	900001	904000	deletion_72	0,142147	4000
scaffold_6	1363001	1365000	deletion_77	0,21486	2000
scaffold_6	1903001	1912000	deletion_80	0,261699	9000
scaffold_7	1673001	1709000	deletion_86	0,016033	36000
scaffold_9	1633001	1638000	deletion_103	0,22327	5000
scaffold_16	1	49000	deletion_142	0,655599	49000
scaffold_17	682001	687000	deletion_154	0,115014	5000
scaffold_23	101001	110000	deletion_174	0,203348	9000
scaffold_23	138001	140000	deletion_175	0,136662	2000
scaffold_23	320001	331000	deletion_177	0,0154556	11000
scaffold_26	22001	37000	deletion_180	0,112214	15000
scaffold_27	1	76000	deletion_182	0	76000
scaffold_28	1	51000	deletion_183	0,249134	51000
scaffold_95	1	12000	deletion_186	0	12000
scaffold_168	1	6000	deletion_188	0,24174	6000
scaffold_180	1	6000	deletion_189	0	6000
scaffold_181	1	6000	deletion_190	0	6000
scaffold_183	1	5000	deletion_191	0	5000
scaffold_193	1	5000	deletion_192	0	5000
scaffold_197	1	5000	deletion_193	0	5000
scaffold_198	1	5000	deletion_194	0	5000
scaffold_205	1	5000	deletion_195	0	5000
scaffold_212	1	4000	deletion_196	0	4000
scaffold_224	1	4000	deletion_197	0	4000
scaffold_228	1	3000	deletion_198	0	3000
scaffold_255	1	2000	deletion_199	0,0886661	2000
scaffold_261	1	2000	deletion_200	0	2000
scaffold_263	1	2000	deletion_201	0	2000
scaffold_296	1	2000	deletion_203	0	2000
scaffold_298	1	2000	deletion_204	0	2000
scaffold_315	1	2000	deletion_205	0	2000
scaffold_317	1	2000	deletion_206	0,105737	2000
scaffold_324	1	2000	deletion_207	0	2000
scaffold_330	1	2000	deletion_208	0	2000
scaffold_331	1	2000	deletion_209	0,0762775	2000
scaffold_333	1	2000	deletion_210	0,142137	2000
scaffold_334	1	2000	deletion_211	0	2000
scaffold_338	1	2000	deletion_212	0	2000
scaffold_339	1	2000	deletion_213	0	2000
scaffold_350	1	2000	deletion_214	0	2000
scaffold_353	1	2000	deletion_215	0,195168	2000
scaffold_359	1	2000	deletion_216	0,123428	2000
scaffold_363	1	2000	deletion_217	0,0328393	2000
scaffold_366	1	2000	deletion_218	0,0233588	2000
scaffold_374	1	2000	deletion_219	0,0123318	2000

Table S3. GO enrichment analysis of the 291 deleted genes.

GO ID	Category	Description	Number in input list	Number in BG/Ref	p-value	FDR	Fold Enrichment
GO:0019207	F	kinase regulator activity	5	6	1,40E-07	1,90E-05	26,57
GO:0019887	F	protein kinase regulator activity	5	6	1,40E-07	1,90E-05	26,57
GO:0008238	F	exopeptidase activity	8	36	9,10E-06	0,00085	7,09
GO:0004180	F	carboxypeptidase activity	5	15	5,40E-05	0,0021	10,63
GO:0051540	F	metal cluster binding	6	24	5,90E-05	0,0021	7,97
GO:0004185	F	serine-type carboxypeptidase activity	5	15	5,40E-05	0,0021	10,63
GO:0070008	F	serine-type exopeptidase activity	5	15	5,40E-05	0,0021	10,63
GO:0051536	F	iron-sulfur cluster binding	6	24	5,90E-05	0,0021	7,97
GO:0008236	F	serine-type peptidase activity	5	39	0,0057	0,16	4,09
GO:0017171	F	serine hydrolase activity	5	39	0,0057	0,16	4,09
GO:0030234	F	enzyme regulator activity	5	57	0,027	0,68	2,80
GO:0050662	F	coenzyme binding	6	231	0,67	1	0,83
GO:0046872	F	metal ion binding	38	1069	0,16	1	1,13
GO:0005488	F	binding	83	3218	0,85	1	0,82
GO:0003676	F	nucleic acid binding	32	1012	0,37	1	1,01
GO:0003677	F	DNA binding	22	688	0,38	1	1,02
GO:0008233	F	peptidase activity	9	182	0,094	1	1,58
GO:0043167	F	ion binding	38	1086	0,18	1	1,12
GO:0001883	F	purine nucleoside binding	19	674	0,6	1	0,90
GO:0001882	F	nucleoside binding	19	675	0,6	1	0,90
GO:0043169	F	cation binding	38	1086	0,18	1	1,12
GO:0046914	F	transition metal ion binding	28	976	0,57	1	0,91
GO:0016462	F	pyrophosphatase activity	10	287	0,34	1	1,11
GO:0016829	F	lyase activity	7	189	0,32	1	1,18
GO:0016407	F	acetyltransferase activity	5	74	0,068	1	2,15
GO:0000166	F	nucleotide binding	31	824	0,12	1	1,20
GO:0016740	F	transferase activity	28	853	0,31	1	1,05

GO:0016746	F	transferase activity, transferring acyl groups	6	138	0,22	1	1,39
GO:0016747	F	transferase activity, transferring acyl groups other than amino-acyl groups	6	121	0,15	1	1,58
GO:0004672	F	protein kinase activity	6	206	0,56	1	0,93
GO:0017076	F	purine nucleotide binding	21	744	0,6	1	0,90
GO:0005506	F	iron ion binding	5	227	0,8	1	0,70
GO:0016491	F	oxidoreductase activity	34	1189	0,58	1	0,91
GO:0005524	F	ATP binding	15	563	0,68	1	0,85
GO:0016787	F	hydrolase activity	28	1172	0,87	1	0,76
GO:0005215	F	transporter activity	18	519	0,28	1	1,11
GO:0016301	F	kinase activity	9	283	0,45	1	1,01
GO:0017111	F	nucleoside-triphosphatase activity	10	273	0,29	1	1,17
GO:0003824	F	catalytic activity	102	4028	0,9	1	0,81
GO:0048037	F	cofactor binding	9	358	0,72	1	0,80
GO:0022891	F	substrate-specific transmembrane transporter activity	8	242	0,42	1	1,05
GO:0016818	F	hydrolase activity, acting on acid anhydrides, in phosphorus-containing anhydrides	10	288	0,34	1	1,11
GO:0022892	F	substrate-specific transporter activity	9	258	0,35	1	1,11
GO:0016614	F	oxidoreductase activity, acting on CH-OH group of donors	7	179	0,28	1	1,25
GO:0030528	F	transcription regulator activity	7	545	1	1	0,41
GO:0016616	F	oxidoreductase activity, acting on the CH-OH group of donors, NAD or NADP as acceptor	5	131	0,34	1	1,22
GO:0046906	F	tetrapyrrole binding	5	176	0,59	1	0,91
GO:0016773	F	phosphotransferase activity, alcohol group as acceptor	6	264	0,79	1	0,72
GO:0016772	F	transferase activity, transferring phosphorus-containing groups	12	386	0,46	1	0,99
GO:0030554	F	adenyl nucleotide binding	19	674	0,6	1	0,90
GO:0008415	F	acyltransferase activity	6	114	0,12	1	1,68
GO:0016817	F	hydrolase activity, acting on acid anhydrides	10	291	0,36	1	1,10
GO:0003700	F	transcription factor activity	6	446	0,99	1	0,43
GO:0022857	F	transmembrane transporter activity	14	287	0,051	1	1,56
GO:0070011	F	peptidase activity, acting on L-amino acid peptides	9	174	0,076	1	1,65
GO:0032559	F	adenyl ribonucleotide binding	15	563	0,68	1	0,85
GO:0022804	F	active transmembrane transporter activity	7	187	0,31	1	1,19
GO:0032555	F	purine ribonucleotide binding	17	630	0,66	1	0,86
GO:0032553	F	ribonucleotide binding	17	630	0,66	1	0,86
GO:0008270	F	zinc ion binding	22	704	0,42	1	1,00

Table S4. Mapping statistics

	Nº of reads
Raw reads	3158689
Filter reads	3109378
Mapped	2818410
Unique mapped	2314336
Unmapped	290968

Table S5. Copy number variation statistics

Window size	1000
Nº of CNV	219
Negative (deletion)	219
Positive (duplication)	0
High quality CNV	55
Genes in CNV	291
NRPS	1
PKs	1

Table S6. ***Aspergillus carbonarius*** **PKS and NRPS genes annotated in the reference genome**

Aspergillus carbonarius **PKS genes**	***Aspergillus carbonarius*** **NRPS genes**
e_gw1.16.5.1	estExt_fgenesh2_pg.C_1_t10324
estExt_fgenesh2_pg.C_160069	estExt_fgenesh2_pg.C_2_t10203
estExt_fgenesh2_pg.C_3_t20188	estExt_fgenesh2_pg.C_3_t20159
estExt_fgenesh2_pg.C_4_t10336	estExt_fgenesh2_pg.C_4_t10461
estExt_fgenesh2_pg.C_5_t20078	estExt_fgenesh2_pg.C_5_t20078
estExt_fgenesh2_pg.C_7_t10163	estExt_fgenesh2_pm.C_120178
estExt_fgenesh2_pm.C_140200	estExt_fgenesh2_pm.C_1_t10378
estExt_fgenesh2_pm.C_150126	estExt_Genemark1.C_120304
estExt_fgenesh2_pm.C_1_t10378	estExt_Genemark1.C_140234
estExt_fgenesh2_pm.C_4_t10377	estExt_Genemark1.C_30660
estExt_fgenesh2_pm.C_6_t10415	estExt_Genemark1.C_50656
estExt_Genemark1.C_170101	estExt_Genewise1.C_10355
estExt_Genemark1.C_50656	estExt_Genewise1.C_1140001
estExt_Genewise1Plus.C_100641	estExt_Genewise1.C_40855
estExt_Genewise1Plus.C_120511	estExt_Genewise1Plus.C_160111
estExt_Genewise1Plus.C_170239	estExt_Genewise1Plus.C_20059
estExt_Genewise1Plus.C_190277	estExt_Genewise1Plus.C_31605
estExt_Genewise1Plus.C_210015	fgenesh_isotigs_kg.10_#_82_#_isotig03071
estExt_Genewise1Plus.C_30299	fgenesh_isotigs_kg.1_#_800_#_isotig14023
estExt_Genewise1Plus.C_51391	fgenesh_isotigs_kg.3_#_407_#_isotig04772
estExt_Genewise1Plus.C_91104	fgenesh_isotigs_kg.4_#_452_#_isotig12148
fgenesh_isotigs_kg. 13_#_4_#_isotig09366	fgenesh_isotigs_kg.6_#_569_#_isotig11054
Genemark1.5570_g	Genemark1.5570_g
Genemark1.5640_g	Genemark1.5640_g
